# **The** ***Helicobacter pylori*** **eradication in the group receiving standard -dose and group continue taking amoxicillin for 4 weeks; a clinical trial study**

**Published:** 2015

**Authors:** Mohammad Javad Ehsani-Ardakani, Meghdad Sedaghat, Gyti Eslami, Hamid Mohaghegh Shalmani

**Affiliations:** 1 *Emam Hossein Hospital, Shahid Beheshti University of Medical Sciences, Tehran, Iran*; 2 *Gastroenterology and Liver Diseases Research Center, Research Institute for Gastroenterology and Liver Diseases, Shahid Beheshti University of Medical Sciences, Tehran, Iran*

**Keywords:** *Helicobacter pylori*, Amoxicillin, Clarithromycin, Omeprazole

## Abstract

**Aim:**

The aim of this study was to evaluate the *Helicobacter pylori* eradication in the group receiving standard -dose twice a day for two weeks and continue taking amoxicillin for 4 weeks.

**Background:**

*Helicobacter pylori* is the major etiological cause of chronic gastritis, gastric and duodenal ulcers, gastric cancer and lymphoma. Therefore, patients should be treated after diagnosis *of H. pylori* infection.

**Patients and methods:**

A total of 66 consecutive patients with rapid urease test during endoscopy or biopsy positive for *H. pylori *were enrolled in this clinical trial study during 2013-2014. Patients were divided randomly into two groups. Group A (standard dose) received omeprazole (20 mg), amoxicillin (1 g), and clarithromycin (500 mg), all two times a day for two weeks. Group B received standard dose like group A and in patients with *H.pylori* infection amoxicillin were continued for 4 weeks. After completion of treatment, patients did not receive any treatment for a month and then stool antigen was performed to evaluate the *H.pylori*.

**Results:**

The rate of successful HP eradication was significantly higher in group A (90.9% V.s 63.6%; p=0.017). Inflation and bitter mouth were found in 8 and 13 patients in group A and 7 and 9 patients in group B, respectively. The incidence of adverse effects was the same (p=0.437).

**Conclusion:**

Increased duration of antibiotic therapy to four weeks significantly raises the rate of successful HP eradication with standard triple therapy without significant increase in adverse effects.

## Introduction

*Helicobacter pylori* (HP) is the most common chronic bacterial infection in humans (1, 2). It is estimated that 60% of the world population is infected with HP infection (1, 3). The incidence of HP infection in the United States, as well as developed and developing countries has been reported between 30% and 80% respectively (4). It is indicated that more than 70% of infected cases are asymptomatic (5). In the United States, its prevalence is influenced by age and approximately 50% of infected cases are around 60 years old and 25% of patients are 30 years old (1). Low incidence of HP infection among children in developed countries is due to improvements in lifestyle and increased use of antibiotics (6). 

The HP infection is prevalent worldwide and associated with many gastrointestinal problems (1, 7-10). In particular, it has been shown that HP infection plays a role in the pathogenesis of peptic ulcers, chronic/active gastritis and gastric cancer (2, 11-22). In this situation patients should be treated if the *H.pylori* infection diagnosed. HP eradication is the main treatment of peptic ulcer disease and other gastroduodenal disorders (16, 23-27).Studies have shown that eradication of HP is accompany by reducing the severity of gastritis and recurrence peptic ulcer rate (3, 12, 28, 29). Appropriate treatment of HP infection is unknown (1). In 1994, NIH (National Institutes of Health) reported that patients with peptic ulcer disease and *H.pylori* infection require treatment with antimicrobial agents (2). Therefore, in the last decades the standard 3-drug regimen including amoxicillin, clarithromycin, and metronidazole are widely used in many countries as a first step regimen for HP eradication (8, 27, 30-38). 

In recent year’s eradication of HP infection in some cases were failed due to increasing resistance to clarithromycin and metronidazole (1-3). In different clinical trials, 61 to 93% eradication rate of *H. pylori* has been reported using two, three and four drugs regimes, whereas in the placebo-control studies eradication rate of *H. pylori* has been shown in 64-69% of cases using two- and three-drug regimen. 

In this study, the eradication rate of *H. pylori* in the two groups, including group A receiving the standard regimen and group B received standard dose like group A and in patients with *H.pylori* infection amoxicillin were continued for 4 weeks were evaluated. 

## Patients and Methods

In this clinical trial, patients with *Helicobacter pylori* infection during the period of 2013-14were recruited the study. Inclusion criteria, ,were including stopping using bismuth or antibiotics within 4 weeks before entry the study, no history of eradication of *H.pylori* or gastric cancer in the endoscopy report, no history of allergy to amoxicillin and omeprazole, no previous gastric surgery. 

Invasive tests and/or rapid urease test were the first to be used in the diagnosis of *H. pylori* infection.

Then the data of the infected patients were recorded during endoscopy. 

Following ethical and research committee approval, subjects gave their informed consent and was recruited into this study. Then the data of patients were collected using a valid questionnaire and divided randomly into two groups. For group A, a standard triple therapy for 2 weeks and then continued with 1000 mg amoxicillin every 12 hours for the next 2 weeks were administered. For group B, standard triple therapy for 2 weeks and then continued placebo every 12 hours for the next two weeks were administered. 

It should be noted that omeprazole/amoxicillin/clarithromycin (Rx) every 12 hours was considered as the standard regimen in this study. After completion of treatment, patients did not receive any treatment for a month and then *H.pylori* stool antigen test was evaluated. Patients with drug intolerance were excluded. Finally, data regarding *H. pylori* eradication after treatment were collected and compared between the two groups. It should be noted that in order to reduce bias in the study, lab technicians were blind to the duration of treatment, dose of the drugs and type of regimen. 

**Statistical analysis**

In this study, non-probability convenience sampling was used. To compare quantitative data between the two groups, independent samples t-test and for qualitative data Fisher's exact test or chi-square test were used. Statistical analyzes were performed using the SPSS software ver.16 and P<0.05 was considered as significant. 

## Results

Out of 80 patients, 14 patients found it hard and difficult to continue and subsequently were withdrawn from study and eventually study was conducted on 66 patients in the two groups. The mean age of patients in group A was 44.6 ± 14.9 years (range 23-77) and in group B was 41.8 ± 14.6 years (range 17-78). The difference between the two groups was not statistically significant (p=0.39). In the group A, 18 cases were men and in the placebo group, 16 were male. Distribution of comorbidities, the main complaint, smoking status and endoscopic findings of patients in both groups were presented in Table 1 and show that there is no significant difference between the two groups (p>0.05).

According to the study, we found that HP infection was successfully eradicated in 30 patients (90.9%) and 21 (63.6%) in group A and group B respectively. In this case, the difference between the two groups was statistically significant (p=0.01).

**Table 1:** Comparison of comorbidities, patient complaints, smoking and endoscopic findings between the two groups

Variable		Group A	Group B	P value
Comorbidities	Without Comorbidities	6	9	0.55
	dyspepsia	17	13	
	GERD	10	11	
Main complication	Epigaster pain	24	21	0.82
	Melena	1	3	
	Difficulty swallowing	0	1	
	Bloating	2	1	
	Nausea	1	1	
	Burning	0	1	
	Heart burn	2	2	
	Bloating	1	2	
	Weight loss	2	1	
Smoking	Active smokers	6	3	0.23
	Passive smokers	2	0	
	Quit smoking	0	1	
	Nonsmokers	25	29	
Endoscopy findings	Normal	11	9	0.51
	Ulcer	2	0	
	Duodenal ulcer	2	1	
	Gastritis	17	21	
	Scrape pulp	1	2	

The only treatment-related side effect seen in the most of patients were bloating and bitter mouth. The incidence of these side effects was similar in the two groups (p=0.437). The main complaint of patients in both groups was epigastric pain, which is presented in 23 and 21 subjects in group A and B respectively. In both groups, the most common endoscopic findings were gastritis. 

## Discussion

The result of this study showed that increase the duration of amoxicillin usage is significantly associated with an increase in the success rate of HP infection eradication. In addition, we found that this method is a safe method and treatment-related side effects are as similar as common methods of treatment. 

Eradication of HP infection is one of the fundamental challenges in GI science. Omeprazole, clarithromycin and amoxicillin regimens are widely used for HP eradication due to high efficacy and limited side effects (28, 39). On the other hand due to unsuccessful treatment of HP infection, researchers recommended the newer therapies such as quadruple therapy. There are several factors that can affect the success rate of HP eradication. One of the most important factors is the increasing antibiotic resistance, which raised these days and knowledge of the antibiotic resistance pattern can greatly assist in providing the most appropriate treatment strategy (40-42). It is very difficult to determine and assess the resistance of HP regularly, because the concentration of the antibiotic should be considered in gastric mucosal not in the blood (40).

Some researchers believe that in spite of using the same treatment regimens with Western countries, the success rate of HP infection eradication is much lower and therefore, the recurrence rate is much higher (43). To control the HP resistance, some researchers suggested increasing the duration of antibiotic treatment. 

For instance, Fallone et al. recommended duration of proton pump inhibitor triple therapy should be increased from seven to 14 days (44). They concluded that an increased duration of treatment with triple therapy with PPI could significantly increase the success rate of HP eradication without being accompanied by an increased incidence of side effects. Also, Greenberg et al. performed randomized trial of experimental 14-day triple, 5-day concomitant, and 10-day sequential therapies for *H. pylori* eradication (45). The result of their trial showed that standard two-week triple-drug therapy is more effective than 5-day concomitant or 10-day sequential four-drug regimens as an experimental therapy for *H pylori* infection. Iwańczak suggested that to obtain better results of the eradication, the treatment should be prolonged from 7 to 10 or even 14 days, and it is necessary to increase the dose of antibiotics (46). However, despite these studies, Yoon and colleagues demonstrated that an increased duration of treatment did not increase the success of HP eradication due to the rapid increase in bacterial resistance. (47)

In the present study, we examined the effect of increasing treatment duration from 14 to 28 days, and success rate of HP eradication. We have observed that increasing the duration of treatment was associated with a significantly increased HP eradication and as a result, successful treatment was detected in approximately 90% of patients in group-A. However, the high percentage of patients with placebo failed to treatment (36.4%) and this may indicate that the increased efficiency of antibiotic therapy is associated with an increased success rate of HP eradication. These findings are in accordance with previous studies that suggest prolonged antibiotic therapy can play an important role in increasing the success rate of HP eradication. This is an effective treatment and has been recommended by authors.
